# The Use and Misuse of Mathematical Modeling for Infectious Disease Policymaking: Lessons for the COVID-19 Pandemic

**DOI:** 10.1177/0272989X21990391

**Published:** 2021-02-03

**Authors:** Lyndon P. James, Joshua A. Salomon, Caroline O. Buckee, Nicolas A. Menzies

**Affiliations:** Harvard University, Cambridge, MA, USA; Center for Health Policy and Center for Primary Care and Outcomes Research, Stanford University, Stanford, CA, USA; Center for Communicable Disease Dynamics, Harvard T. H. Chan School of Public Health, Boston, MA, USA; Department of Global Health and Population, Harvard T. H. Chan School of Public Health, Boston, MA, USA

**Keywords:** COVID-19, infectious diseases, mathematical modeling, uncertainty, validation

## Abstract

Mathematical modeling has played a prominent and necessary role in the current coronavirus disease 2019 (COVID-19) pandemic, with an increasing number of models being developed to track and project the spread of the disease, as well as major decisions being made based on the results of these studies. A proliferation of models, often diverging widely in their projections, has been accompanied by criticism of the validity of modeled analyses and uncertainty as to when and to what extent results can be trusted. Drawing on examples from COVID-19 and other infectious diseases of global importance, we review key limitations of mathematical modeling as a tool for interpreting empirical data and informing individual and public decision making. We present several approaches that have been used to strengthen the validity of inferences drawn from these analyses, approaches that will enable better decision making in the current COVID-19 crisis and beyond.

## Mathematical Modeling and Coronavirus Disease 2019

Since the emergence of coronavirus disease 2019 (COVID-19) as a global pandemic, many policymakers have relied on mathematical models to guide highly consequential decisions about mitigation strategy, balancing the goals of protecting health while limiting economic and social disruption. This endeavor can be especially challenging when models disagree. For example, a model from the Institute for Health Metrics and Evaluation (IHME) in April 2020 forecast around 60,000 total deaths from COVID-19 in the United States during the first wave of the pandemic.^1^ This figure was passed before the end of April, with more than 125,000 confirmed COVID-19 deaths reported by July 1, at the end of the first wave.^2^ The IHME model was reportedly influential in White House deliberations over strategy,^3^ even as epidemiologists and modelers criticized its projections as overly optimistic and methodologically flawed.^4–7^ IHME has since made several major revisions in response to such criticism,^8^ and their recent analyses have projected more than 500,000 deaths by March 2021, similar to other prominent models. The IHME model is hardly the only model to be received with skepticism.^9–11^ Early in the pandemic, several models offered starkly different projections for COVID-19 cases and deaths,^12^ and months later, differences between the available models still persist.^13^

Although these publicized disagreements may have contributed to public mistrust of mathematical modeling,^7^ models remain essential tools for evidence synthesis, planning and forecasting, and decision analysis for infectious disease policymaking. They enable formal and explicit consolidation of scientific evidence on the many factors relevant to a decision, and allow analysts to estimate dynamic outcomes that would be difficult or impossible to measure empirically, including the long-term consequences of policy alternatives. Given the high level of uncertainty around many important parameters (such as the level and duration of immunity to COVID-19, the duration of the latency and incubation periods, and adherence to physical distancing, mask wearing, and other mitigation measures), mathematical models can be used to explore uncertainties around model inputs and assumptions, as well as project plausible ranges for each outcome of interest. These characteristics make models highly valuable planning tools. By identifying the assumptions and uncertainties to which decision making is most sensitive, they can also be used to prioritize research investments, describing the information that is most important to collect to allow better decision making.^14^

In the COVID-19 pandemic, prominent modeling applications have been used to chart out possible worst-case scenarios,^15,16^ shape decisions around major policies such as physical distancing^9,17,18^ and testing,^19,20^ plan for the deployment of public health resources,^21–25^ and infer key epidemiological parameters describing how the epidemic might manifest in different settings.^9,26^ These different purposes shape decisions about model complexity and approach, the level of precision required of model results, and the extent to which modeling conclusions will generalize to different situations or questions.

## The Challenges of Modeling

In all cases, analysts constructing mathematical disease models make decisions about how to represent partially observed processes—such as disease natural history or how the public will respond to a new disease threat—that generate the consequences and outcomes of interest. Due to imperfect mechanistic information, there can be multiple defensible approaches for constructing and parameterizing models, all consistent with current evidence, but that may nevertheless diverge in their future predictions. Sensitivity of results to these design choices complicates the interpretation of modeling studies. This interpretation has been made more challenging in the current pandemic, with rapid changes in the evidence base and the pressing demand for definitive answers from the public and policymakers. However, concerns over the validity of modeling studies have long existed, and the current debate about severe acute respiratory syndrome coronavirus 2 (SARS-CoV-2) modeling mirrors earlier discussions in other disease areas.

**Figure 1:**
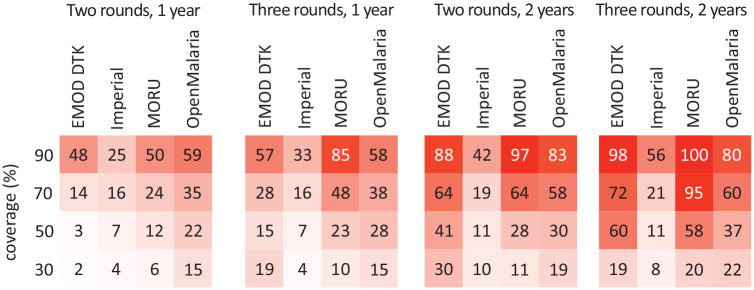
The percentage reduction in the all-age prevalence of *Plasmodium falciparum* malaria in the third year after mass drug administration, as predicted by 4 different mathematical models (EMOD Disease Transmission Kernel [DTK], Imperial, Mahidol Oxford Tropical Medicine Research Unit [MORU], Open Malaria), under 4 coverage scenarios and 4 administration strategies. Re-created from figure in Brady OJ, Slater HC, Pemberton-Ross P, et al. Role of mass drug administration in elimination of *Plasmodium falciparum* malaria: a consensus modelling study. *Lancet Glob Health*. 2017;5(7):e680–7.

Most modeling studies report results from a single model, either by using fixed parameter values or by averaging the results of multiple parameter sets.^27^ Although estimates of uncertainty are sometimes presented, these are typically used to show the stochastic variation in epidemic trajectories or the range of results produced with alternative parameter values. However, when several models analyze the same question, there can be large differences in reported estimates not attributable to stochastic or parametric uncertainty alone but to the modeling approach adopted, modeling structural decisions, and how empirical evidence is incorporated into the model. For example, to evaluate the effectiveness of mass drug administration for malaria control, Brady et al.^28^ compared the expected reductions in malaria prevalence using 4 well-established mechanistic models, all calibrated to the same transmission setting and examining the same 16 intervention scenarios. As shown in Figure 1, while the models largely agreed on the ordering of interventions, they diverged enormously in terms of the effect size of any particular intervention and of the incremental benefits of one intervention compared to another, differences that would be critical to decision makers from a benefit-harm or cost-effectiveness standpoint.

**Figure 2:**
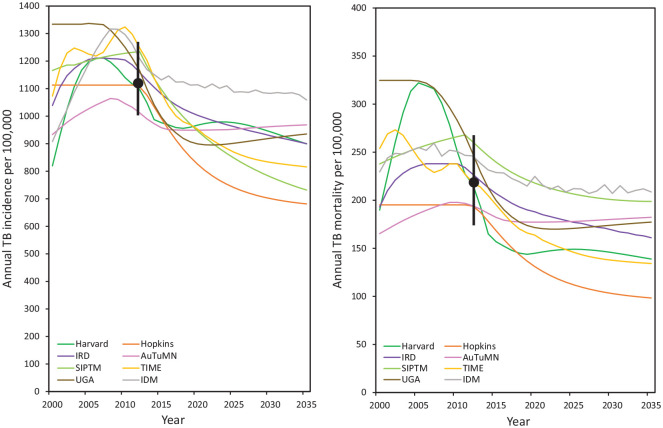
The left panel shows projected tuberculosis (TB) incidence rates in South Africa over the period from 2000 to 2025 from 8 independent models (Harvard, Institut de Recherche pour le Développement [IRD], Stanford [SIPTM], University of Georgia [UGA], Johns Hopkins [Hopkins], Australian Tuberculosis Modelling Network [AuTuMN], London School of Hygiene and Tropical Medicine/Futures/TB Modelling and Analysis Consortium [TIME], Institute for Disease Modeling [IDM]). The black dot and error bar represent the calibration target point estimate and range, respectively. All models were calibrated to the target range for 2012. The right panel shows calibration target and model projections for TB mortality rates over 2000 to 2025. The calibration target (range) for TB incidence was 1117 per 100,000 per year (1002, 1259). The calibration target (range) for TB mortality was 220 per 100,000 per year (179, 270). Adapted from figure in Houben RM, Menzies NA, Sumner T, et al. Feasibility of achieving the 2025 WHO global tuberculosis targets in South Africa, China, and India: a combined analysis of 11 mathematical models. *Lancet Glob Health*. 2016;4(11):e806–15.

Even if there is agreement at a single point in time, models may diverge in their predictions over other time periods. To illustrate, in an analysis of tuberculosis incidence and mortality over 2000–2025 in South Africa, Houben et al.^29^ reported substantial divergence between disease trends produced by 8 independently developed models, as shown in Figure 2. Even though models had been calibrated to fall within prespecified intervals for 2012, the comparison revealed great variation in modeled disease trends. Models reporting steep increases in incidence provided a different view of the epidemiological situation compared to models predicting a steady state or those showing declines.

**Figure 3:**
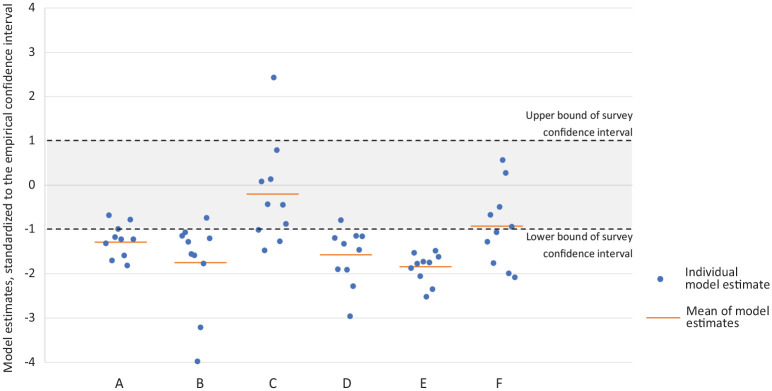
The comparison of predicted outcomes from 10 transmission-dynamic human immunodeficiency virus (HIV) models in South Africa and subsequently available national survey data from 2012. The outcomes are (A) HIV prevalence for adults aged 15 to 49, (B) HIV prevalence for women aged 15 to 49, (C) HIV prevalence for men aged 15 to 49, (D) ratio of women to men covered by antiretroviral therapy, (E) HIV incidence rate for women aged 15 to 49, and (F) HIV incidence rate for men aged 15 to 49. For each outcome, the figure shows the difference between the survey value and model estimates, scaled to the width of the survey confidence interval (shaded gray). Individual model estimates are shown as blue points, and the simple mean of these estimates is shown with an orange line. Adapted from figures presented in Eaton JW, Bacaër N, Bershteyn A, et al. Assessment of epidemic projections using recent HIV survey data in South Africa: a validation analysis of ten mathematical models of HIV epidemiology in the antiretroviral therapy era. *Lancet Glob Health*. 2015;3(10):e598–608.

In some rare examples prior to COVID-19, model results have been checked against later empirical data. Using data from South Africa, Eaton et al.^30^ reported on a comparison between 10 modeled forecasts of human immunodeficiency virus (HIV) prevalence and treatment coverage and the findings of a subsequent national survey that reported the same outcomes. Figure 3 summarizes these contrasts—while for some outcomes, the model estimates were distributed around the survey mean, for others, the estimates were *systematically* different, with most or all modeled estimates falling to one side of the survey confidence interval. Thus, while aggregating the results of multiple models may reduce the impact of misspecification by any single model, pooled results will still be sensitive to any systematic biases.

Such modeling biases are more likely when evaluating policies in novel and rapidly evolving epidemiological circumstances, such as those being considered for COVID-19 control. For established policies and interventions, accumulated evidence will document the realities of routine implementation, whereby policy impact can be less than originally envisaged^31^ and can sometimes be harmful.^32^ For new policies, these factors that limit effectiveness may not be well described and harmful unintended consequences not yet known. This may not be helped by overreliance on early trials, which are typically conducted in populations where greater impact is expected and where interventions are provided with a level of fidelity impractical in routine health services.^33,34^ Together with publication bias, failure to rigorously monitor and validate interventions, and the conscious or unconscious advocacy of well-meaning researchers, systematic biases in the modeling of novel policies can overestimate the likely impact of these policies and systematically bias policymakers in their favor.

## Ways to Identify and Address Modeling Biases

As use of mathematical models has become more commonplace, approaches have evolved to guard against modeling biases. First, individual studies may explore how different modeling assumptions affect their projections.^35^ Another approach, somewhat akin to systematic reviews, is that of model comparison studies, including some examples mentioned above. In these studies, researchers compare projections from multiple models and examine how any differences are related to modeling assumptions. Recently, Drolet et al.^36^ conducted a review of 115 such model comparison studies for vaccine-preventable diseases. They found that, while methodological heterogeneity made it difficult to draw quantitative conclusions, these studies were valuable for identifying tenets of good practice in modeling. Guidelines have been proposed to standardize the process of model comparison,^37^ and within some disease areas, consensus guidance has been developed on good modeling practices.^38^

A separate line of methodological research has examined the biases associated with parameter inference using mechanistic computer models. This work demonstrates that failures to account for model discrepancy^39,40^—an imperfect fit between model outcomes and the data used to fit them—may lead to parameter estimates and model predictions that are overly precise and systematically biased. The relevance of model discrepancy for health policy analysis has also been explored.^41^ While formal approaches have been developed to account for imperfect fit between model and calibration data,^42,43^ these methods have infrequently been used in infectious disease modeling.

Many of the considerations discussed above pertain to how a model’s outcomes are validated. However, the value of such validation depends critically on the nature of the data available for validation. As shown in Figure 2, models with different assumptions can produce remarkably similar outcomes at certain points in time while diverging at others. This partial consistency—which may be used to argue that models agree—is irrelevant if the crucial policy questions relate to the time period where results diverge. Similarly, justifying a model’s fitness for purpose by validating it against current policy outcomes is insufficient if divergent results are seen when models are used to forecast the results of a *different policy* under consideration. This issue—that the model outcomes needed for decision making differ from the outcomes that can be compared to other evidence—complicates the task of model validation and renders approaches like cross-validation^44^ less relevant for policy modeling. As it is generally never possible to validate all outcomes of interest (otherwise, why is a model being used at all?), there will always be some assumptions needed. Blanket claims of the “validity” of a model should be viewed with suspicion.

Even if the validation of model *outcomes* is difficult, it is still possible to interrogate model *processes*. One advantage of mechanistic models (as compared to purely statistical models) is that they attempt to reproduce the underlying processes that generate observed outcomes, such as disease natural history or the processes of providing health care. Because these intermediate calculations are designed to represent real, physical processes, the structures and parameters used to model these mechanisms can be critiqued and compared to external data. For example, in a 2018 systematic review of over 300 tuberculosis transmission models,^45^ huge variation in modeled disease risks was attributed to differences in the representation of latent disease, a crucial part of TB natural history. Critically, all of these models could be calibrated to reproduce a particular incidence and mortality profile but would produce very different results if used to compare policy options. By comparing modeled disease risks to empirical data, models that are inconsistent with these data can be identified.

Model benchmarking and validation are frequently undertaken by multimodel collaborations. In the United States, the Centers for Disease Control and Prevention (CDC) curates a set of (to date) 37 COVID-19 forecasting models developed by independent research teams.^46^ These models have been compared against each other and validated against reported data, as well as used to project future hospitalizations and deaths. An ensemble model has also been developed to combine the participating models.^12^ Comparable to a meta-analysis, an ensemble model aims to improve predictive performance by calculating a weighted average of the results of several models, each of which may rely on different assumptions and data.^47,48^ Weights are typically chosen to minimize prediction error of the ensemble,^49^ but alternative weighting schemes can prioritize other features if desired. This COVID-19 ensemble provides forecasts for each US state, and most of the component models are mechanistic in nature. It has offered projections since early April, and the true number of deaths for the United States has mostly fallen within the model’s 95% credibility interval. Other collaborations and repositories are also being established to document the COVID-19 models that are being developed and will facilitate later comparisons.^50,51^ The rapid accumulation of empirical data will provide greater opportunities for model validation early in the model development process, which may be enhanced by the adoption of data assimilation frameworks.^52,53^ In settings of rapid epidemiological change, validation may only be possible after modeled results are in the public domain. As such, use of models for real-time decision making can be perilous, as with the influence of early IHME forecasts over White House decision makers, despite prominent critiques of the model.^4–7,54^

## Living with Modeling Uncertainty

In the rapidly evolving climate of the COVID-19 pandemic, there are major uncertainties around disease dynamics and policy outcomes, as well as ample opportunity for models to “get it wrong.” We should expect that the evidence base and epidemiological context will continue to shift, sometimes making earlier modeled results obsolete. Modeling is likely to remain prominent as new policy questions arise, yet the uncritical acceptance of modeling results will not serve public health or the field of modeling. Careful evaluation and comparison of results—and benchmarking against empirical findings where possible—will be important for revealing assumptions and potential biases, as well as spurring progressive improvement in modeling approaches.
